# CHildren of the Cohort Study (CHOC): Exploring parenting desire among people living with HIV in Switzerland

**DOI:** 10.1111/hiv.70253

**Published:** 2026-05-11

**Authors:** Clara Merlin, José Damas, Olivier Nawej Tshikung, Noémie Wagner, Anna Hachfeld, Andrea Duppenthaler, Marcel Stoeckle, Malte Kohns, Irene A. Abela, Paolo Paioni, Luigia Elzi, Lisa Kottanattu, Patrick Schmid, Christian R. Kahlert, Katharina Kusejko, Pierre Alex Crisinel, Pascal Pellegrino, Matthias Cavassini, Katharine E. A. Darling, the Swiss HIV Cohort Study, IA Abela, K Aebi‐Popp, A Anagnostopoulos, E Bernasconi, DL Braun, HC Bucher, A Calmy, M Cavassini, A Ciuffi, G Dollenmaier, M Egger, L Elzi, JS Fehr, J Fellay, S Frigerio Malossa, CA Fux, HF Günthard, A Hachfeld, DHU Haerry, B Hasse, HH Hirsch, M Hoffmann, I Hösli, M Huber, D Jackson‐Perry, CR Kahlert, D Kaufmann, O Keiser, T Klimkait, RD Kouyos, H Kovari, K Kusejko, ND Labhardt, K Leuzinger, B Martinez de Tejada, C Marzolini, KJ Metzner, N Müller, J Nemeth, D Nicca, J Notter, P Paioni, Child Substudy, G Pantaleo, M Perreau, A Rauch, LP Salazar‐Vizcaya, P Schmid, O Segeral, RF Speck, M Stöckle, B Surial, PE Tarr, A Trkola, G Wandeler, M Weisser, S. Yerly

**Affiliations:** ^1^ Infectious Diseases Service University Hospital Lausanne, University of Lausanne Lausanne Switzerland; ^2^ Division of Infectious Diseases University Hospital Geneva, University of Geneva Geneva Switzerland; ^3^ Hôpital des Enfants University Hospital Geneva, University of Geneva Geneva Switzerland; ^4^ Department of Infectious Diseases Bern University Hospital, University of Bern Bern Switzerland; ^5^ Department of Paediatrics, Unit of Paediatric Infectious Diseases Bern University Hospital, University of Bern Bern Switzerland; ^6^ Division of Infectious Diseases and Hospital Epidemiology University Hospital Basel, University of Basel Basel Switzerland; ^7^ University Children's Hospital University of Basel Basel Switzerland; ^8^ Department of Infectious Diseases and Hospital Epidemiology University Hospital Zurich, University of Zurich Zurich Switzerland; ^9^ Institute of Medical Virology University of Zurich Zurich Switzerland; ^10^ Division of Infectious Diseases and Hospital Epidemiology University Children's Hospital Zurich Switzerland; ^11^ Division of Infectious Diseases Regional Hospital Lugano Lugano Switzerland; ^12^ Institute of Paediatrics of Southern Switzerland, EOC Bellinzona Switzerland; ^13^ Faculty of Biomedical Science Università della Svizzera Italiana Lugano Switzerland; ^14^ Division of Infectious Diseases, Infection Prevention and Travel Medicine, HOCH Health Ostschweiz, Cantonal Hospital St. Gallen University Teaching and Research Hospital St. Gallen Switzerland; ^15^ Infectious Diseases and Infection Prevention Children's Hospital of Eastern Switzerland St. Gallen Switzerland; ^16^ Unit of Paediatric Infectious Diseases and Vaccinology, Service of Pediatrics, Department Woman‐Mother‐Child Lausanne University Hospital and University of Lausanne Lausanne Switzerland; ^17^ Author, “Papa Gay”, Editions Favre 2009

**Keywords:** family planning, fertility, HIV influence, parenthood, parenting desire, people living with HIV

## Abstract

**Background:**

Although HIV is now a chronic condition with an excellent life expectancy, some personal aspects affecting people living with HIV (PLWH), such as parenting desire, are relatively understudied. This study aimed to explore parenting desire among PLWH in Switzerland.

**Methods:**

From September 2022 until January 2024, PLWH (participants) enrolled in the Swiss HIV Cohort Study (SHCS) and followed up at seven university/cantonal hospitals were invited to complete a structured questionnaire co‐designed with patient and public involvement. There were no exclusion criteria. The questionnaire covered parenting desire, the perceived influence of HIV on family planning and whether future parenthood had ever been discussed with an HIV physician.

**Results:**

Of the 5038 PLWH attending SHCS clinic visits, 3006 (59.7%) completed the questionnaire (2974 (98.9%) complete datasets analysed). The median age was 54 years (IQR 44;61), 27% were female, 39% were men who have sex with men (MSM), 16% were of Black ethnicity, 94.5% had HIV RNA plasma viral loads <50 copies/ml. Overall, 515 participants (17.3%) expressed a desire for parenting, with a desire associated with being aged <45 years and of Black ethnicity, and 506 (17%) stated that HIV influenced family planning decisions. Discussing parenthood was associated with being female, Black, heterosexual and in a stable relationship. Among heterosexual male participants, 26.1% had ever had such discussions, compared to 6.8% of MSM.

**Conclusion:**

In this sample of PLWH, a minority expressed parenting desire, possibly due to high median participant age, and 17% felt HIV influenced family planning decisions. While female participants were more likely to have discussed future parenthood than male participants, there was a further disparity between heterosexual men and MSM. Whilst surrogacy is prohibited in Switzerland, MSM may have access to parenthood in other ways. Our findings suggest there is room for HIV physicians to proactively invite parenting discussions with all PLWH.

## INTRODUCTION

Modern antiretroviral therapy (ART), resulting in improved health and a near‐normal life expectancy, has enabled more people living with HIV (PLWH) to consider having children. The rate of vertical HIV acquisition has been reduced to less than 1% [1], and vaginal delivery and breastfeeding are now possible for women with undetectable plasma HIV RNA viral loads [2, 3].

In 2008, the Swiss Statement described zero risk of HIV acquisition through condomless sex with PLWH who have undetectable plasma HIV RNA viral loads [4]. On the basis of accumulating evidence [5] this stance was subsequently endorsed and enabled the undetectable equals untransmittable (U=U) campaign to be launched in 2016. PARTNER studies 1 and 2 further proved the absence of risk through condomless sex in sero‐different heterosexual and men same‐sex couples when the HIV‐positive partner was taking suppressive ART [6, 7]. Most studies on family planning conducted among PLWH have been conducted early in the U=U era or in low‐ and middle‐income settings [8, 9]. Parenting desire is complex, influenced by many individual demographic, economic, health‐related, stigma‐associated and socio‐cultural factors [10]. From interviews conducted with 60 PLWH in Tanzania, Pollard et al. found that having children enabled them to conform to social expectations, thus restoring social respectability, a sense of normality and optimism for the future [11].

Several barriers can hinder the desire to have children. Pollard et al. reported the effect of declining health as an economic factor, which can result in a reduced ability to earn and provide for families [11]. Before the U=U era, participants in both low‐ and high‐income countries cited the fear of transmitting HIV to a child or partner, and the anticipated fear of disapproval towards PLWH planning families [11, 12].

Parenting desire is relatively underexplored in Switzerland. In one study conducted among 3023 women enrolled in the Swiss HIV Cohort Study (SHCS), Hachfeld et al compared condom use and pregnancy rates before and after the Swiss Statement [13]. The authors observed an increase from 25% to 75% of condomless sex but without an increase in pregnancy rates, assumed to be related to an ageing female population. They also observed increasing numbers of spontaneous and induced abortions, with the latter rate being twice as high as in the general Swiss population. However, it was not possible in this retrospective analysis to explain whether this was due to ineffective contraception or other reasons [13].

A significant proportion of heterosexual men and men who have sex with men (MSM) living with HIV have been observed to express a desire for parenting in two Canadian studies [10, 14]. In Switzerland, the Federal Act on Medically Assisted Reproduction (*Loi fédérale sur la procréation médicalement assistée*, LPMA) prohibits oocyte donation and surrogacy (LPMA, Art. 4 al. 2–3) [15]. Sperm donation enables infertile heterosexual and lesbian couples to access parenthood (LPMA Art. 3 al. 1) but excludes men living with HIV (MLWH). Indeed, the LPMA (articles 19 and 4) and its associated ordinance (OPMA, Article 7) stipulate that sperm donors must be free of communicable diseases [15, 16]. Same‐sex couples have been permitted to adopt since 2022, following the legalization of same‐sex marriage [17].

While parenthood is possible medically for PLWH, the subject is not routinely broached during HIV clinic visits in Switzerland. The CHildren Of the Cohort (CHOC) study aimed to assess: (1) the proportion of PLWH in Switzerland who desire to have children, (2) the influence of HIV diagnosis on family planning and (3) the proportion of PLWH who have discussed future parenthood with their HIV physician, and how these are related to individual demographic, domestic and socioeconomic characteristics.

## METHODS

### Ethics statement

The Swiss HIV Cohort Study (SHCS) was approved by the local ethics committees of all participating study sites [18]. All SHCS participants gave written informed consent prior to enrolment.

### Patient and public involvement

From the conception of study, patient and public involvement (PPI) took place as a collaboration between Lausanne‐based physicians (KEAD, MC and CM) and an older parent with lived experience of HIV. We also included an author with a personal and professional interest in parenthood in the LGBTQ community. This collaboration involved the steps of the study design, questionnaire creation, results presentation and manuscript review.

### Study setting

The CHildren Of the Cohort (CHOC) study was conducted as a cross‐sectional multicentric study nested in the SHCS. The SHCS is a multicentre and prospective cohort established in 1988, which includes close to 80% of PLWH who receive ART in Switzerland and are followed in one of five University hospitals (Basel, Bern, Geneva, Lausanne, Zurich), two cantonal hospitals (Lugano, St Gallen), 15 affiliated hospitals or by a registered private physician. Sociodemographic, clinical, laboratory and behavioural data are prospectively recorded at registration and every 6 months thereafter using standardized protocols (https://www.shcs.ch/about-shcs/study-design/protocol/). Data quality and consistency are ensured by quality checks and regular visits at participating centres. The reporting of the study follows the Strengthening the Reporting of Observational Studies in Epidemiology (STROBE) guidelines [18]. SHCS‐enrolled PLWH who took part in the current study are referred to here as participants.

### Study design

This study was limited to the five university and two cantonal hospitals affiliated to the SHCS as physicians in these settings could anticipate and accommodate the additional time required to complete the study questionnaire during medical consultations.

### Participants

All adult PLWH enrolled in the SHCS and followed up at one of the five university or two cantonal hospitals were eligible for inclusion. Other than follow‐up outside these hospital centres, there were no exclusion criteria.

### Questionnaire

As no tailored questionnaires for this study existed, two sets of questions were created with PPI (Supplementary material 1). The first question set quantified children born to SHCS participants, the results of which are presented in another publication [19]. The second question set explored participants' parenting desires, influence of HIV on family planning and whether future parenthood had been discussed with their HIV physician. Specifying the HIV physician when discussing future parenthood was based on the fact that it is standard practice within the multidisciplinary teams at the hospital centres in this study for issues discussed with nurses or other non‐medical healthcare professionals to be relayed, with the patients' consent, to the treating physician. It is then the physician's role to broach this issue during the medical consultation. A pilot study to assess questionnaire feasibility was conducted between September 1 and November 30, 2022, among 196 participants attending Lausanne University Hospital. Questionnaires were completed in French by the treating HIV physician with each participant during one of the biannual follow up visits with interpreter assistance if required. Following the pilot study, questions judged as repetitious by participants and/or HIV physicians were removed, and ambiguous questions were reformulated. The adapted questionnaire was translated and back‐translated into the three other main languages used in Switzerland (English, German and Italian) by healthcare professionals (doctors and/or nurses) working in the field of HIV who were bilingual to a native level. An electronic version of the questionnaire in French, English, German and Italian was then embedded in the SHCS follow up platform. It is quite common for such questionnaires to be embedded within the SHCS platform and PLWH attending a follow‐up visit are invited to participate without obligation.

### Intervention

Following the pilot phase, the study was opened to all seven hospital centres between March 1st 2023 and January 9th 2024. As the study questions on parenting desire, family planning and discussing parenthood were retained following the pilot phase, the 196 pilot‐phase participants were included in the study analysis. Prior to completing the questionnaire, the treating physician explained that the objective of the questionnaire was to quantify parental responsibilities which may not be taken into account during the standard SHCS follow‐up questions and thus to improve clinical care.

### Outcomes

Parenting desire was assessed with the statement, ‘I would like to become a parent in the future’, with four response options: strongly agree, agree, disagree and strongly disagree. HIV influence on family planning was assessed with the question, ‘How much does your HIV diagnosis influence family planning?’ with response options: a lot, a bit, not really and not at all. Parenthood discussions with an HIV physician were assessed with the question, ‘Have you ever discussed becoming a parent with your treating HIV doctor?’ with response options: yes, no. For analysis, responses regarding parenting desire and HIV influence on family planning were dichotomized into agree/disagree and influence/do not influence, respectively. This choice was justified by the high skewness of the raw data, which prevented the use of the full scale in univariate tests due to insufficient subgroup sizes.

The above quantitative data were then analysed against participant demographic characteristics (age, ethnicity, education level, sexual preference, marital status, living situation), baseline clinical parameters (date of enrolment in the SHCS, date of HIV diagnosis, probable mode of HIV acquisition, nadir CD4 count, co‐infections and AIDS‐defining diagnoses), and current clinical information (CD4 count and viral load, alcohol and other substance use, diagnosed depression and other comorbidities). Incomplete questionnaires were excluded from the analysis.

### Statistical analyses

Descriptive analyses presented the results as median values (interquartile range, IQR) for continuous variables, and as percentages for categorical variables. Univariable and multivariable logistic regression models were used to evaluate factors associated with the outcomes of interest. Multivariable models were built by adding the variables (clinical, demographic and other characteristics) of interest without an automated selection procedure. Statistical significance was set at a *P* value ≤0.05. The final model was selected based on Akaike and Bayesian Information criteria. All analyses were conducted using Stata SE 18.0 (StataCorp, College Station, Texas, USA).

## RESULTS

Of 9468 PLWH enrolled in the SHCS during the study period, 5038 had at least one clinic visit at one of the seven hospital centres, of whom 3006 (59.7%) completed the questionnaire. The questionnaires of 32 participants were excluded from analysis (missing data), leaving a final study population of 2974 participants. On average, participants completing the questionnaire were more likely to be White, employed and have higher CD4 counts than PLWH who did not participate. When compared to all PLWH enrolled in the SHCS cohort, participants were more likely to be younger, heterosexual, employed or retired, live with friends or in a community, have higher CD4 counts, to have a diagnosis of depression and have co‐morbidities. The median participant age was 54 years (IQR 44–61). Participant demographic, social and clinical characteristics are presented in Table 1.

**TABLE 1:** Participant characteristics.

	*n* (%) or median [IQR]
Sex^a^	
Female	808 (27%)
Male	2166 (73%)
Transgender^b^	20 (1%)
Ethnicity^a^	
White	2216 (75%)
Black	477 (16%)
Hispano‐American	131 (4%)
Asian	139 (5%)
Other/unknown	11 (0.3%)
Education^a^	
Up to mandatory	761 (26%)
Apprenticeship	1237 (42%)
Higher education	560 (19%)
University degree	416 (14%)
Employment^c^	
Yes	1915 (64%)
No	593 (20%)
Retired	466 (16%)
Sexual preference^a^	
Men having sex with Men	1328 (44.65%)
Heterosexual male	838 (28.18%)
Heterosexual female	808 (27.17%)
Living situation^c^	
Alone	1256 (42%)
With spouse/partner	1085 (36%)
With family/children	486 (16%)
With friends/community	111 (4%)
Institution	36 (1%)
Relationship status^c^	
No steady partnership	1214 (41%)
Steady partnership	1753 (59%)
Declining to answer	7 (0%)
Probable HIV acquisition mode^a^	
MSM	1323 (44%)
Transgender	10 (1%)
Heterosexual female	643 (22%)
Heterosexual male	556 (19%)
IVDU female	75 (3%)
IVDU male	154 (5%)
Other	203 (7%)
Years since HIV diagnosis^a^	17 [10;25]
Years since ART start^a^	14 [9;22]
CD4 count (cells/ μL), median [IQR]^c^	705 [525;923]
CD4 count nadir count (cells/ μL), median [IQR]^c^	222 [106;350]
Detectable plasma viral load (>50 cells/μL)^c^	185 (6.2%)
History of AIDS defining illness^c^	658 (22%)
Depression^c^	
No	2632 (89%)
Yes	342 (11%)
Other Comorbidities^c^, ^d^	
No	1843 (62%)
Yes	1131 (38%)

Abbreviations: IQR, interquartile ratio; IVDU, intravenous drug use; MSM, men who have sex with men.

^a^ Recorded at Swiss HIV Cohort Study (SHCS) registration.

^b^ Transgender participants were identified through another SHCS publication [20].

^c^ Taken from the most recent SHCS dataset.

^d^ Cardiovascular, Metabolic diseases/procedures, Liver, Kidney diseases/procedures, Bone and other.

In total, 1151 (38.7%) participants had at least one child, including 54 MSM (4.7%), with a median of one child per parent (IQR 1–2). People with children were more likely to be women, bisexual or heterosexual, of Black ethnicity, to be in a steady partnership and to live with their family and children. Of participants with children, 69.2% became parents after their HIV diagnosis. For those with children born before their HIV diagnosis, the median time before diagnosis was 4 years (IQR 1–9).

Only 515 (17.3%) participants expressed a parenting desire. The prevalence of parenting desire was similar between heterosexual men and women at 21% and 20.1% respectively, but significantly lower among MSM participants at 13.4% (Χ^2^ (2) = 26.81, *p* < 0.001) (Supplementary material 2, Figure S1). As age increased, parenting desire declined, dropping from 66.8% of adults aged <30 years to 25% of those aged >50 years. Across all age categories, participants of Black ethnicity reported a higher parenting desire compared to those of White ethnicity (Supplementary material 2, Figure S2).

Regarding HIV diagnosis influence on family planning, 17% of all participants and 34.9% (180/515) of those who reported a parenting desire said they felt somewhat or very influenced (Supplementary material 2, Figure S3). Women felt proportionally more influenced by an HIV diagnosis than heterosexual men and MSM (24.6% versus 21% and 9.8%, respectively) (Supplementary material 2, Figure S3). HIV diagnosis influence on family planning was higher among Black participants (Supplementary material 2, Figure S4).

According to their recollections, only 631 (21.2%) participants had ever discussed future parenthood with their HIV physician. While 39.9% of all women reported having discussed parenthood with their HIV physician, the percentage was 26.1% among heterosexual men and 6.8% among MSM (Supplementary material 2, Figure S5). Discussed parenthood was reported more frequently among Black compared to non‐Black participants across all age categories (Supplementary material 2, Figure S6).

**FIGURE 1:**
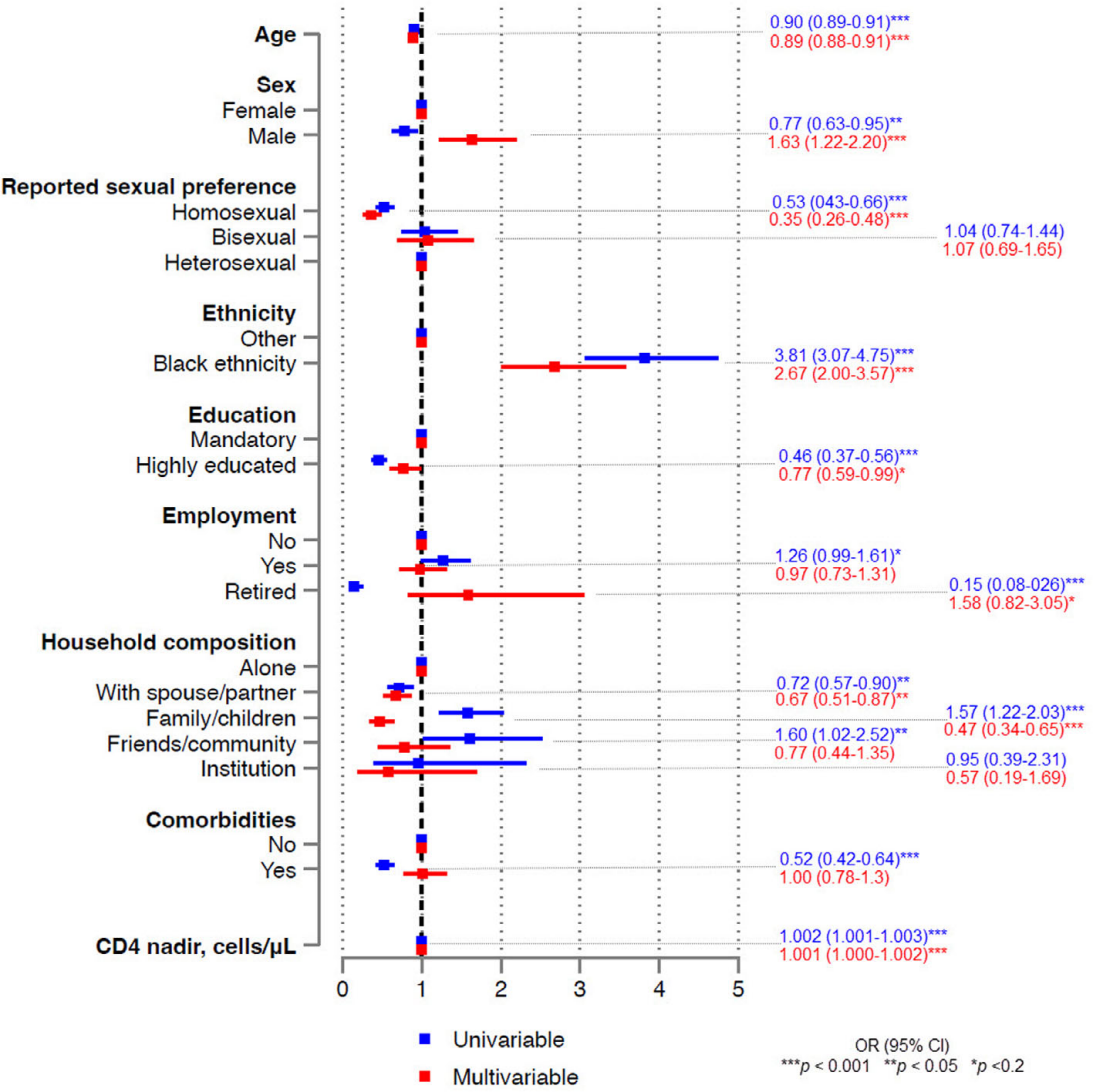
Forest plot of factors associated with parenting desire: Univariate and multivariate logistic regressions with odd ratios and 95% confidence intervals. OR, odd ratio; 95% CI, 95% confidence intervals; *p, p*‐value.

Parenting desire was examined using a multivariate logistic regression model including age, sex, sexual preference, ethnicity, education, employment, living situation, comorbidities and CD4 nadir. The model demonstrated high statistical significance (*n* = 2974, Χ^2^ (14) = 684.31, *p* < 0.001) and a strong fit. The included predictors explained approximately 25% of the variance, although not all remained significant individually (Figure 1). Reduced parenting desire was associated with increasing age (OR 0.89, 95% CI 0.88–0.91, *p* < 0.001), being MSM (OR 0.35, 95% CI 0.26–0.49, *p* < 0.001), having higher education (OR 0.77, 95% CI 0.59–0.99, *p* = 0.047), living with a partner (OR 0.67, 95% CI 0.51–0.87, *p* = 0.003) or with a child (OR 0.47, 95% CI 0.34–0.65, *p* < 0.001). Increased desire was associated with being male (OR 1.63, 95% CI 1.22–2.2, *p* = 0.001) and of Black ethnicity (OR 2.67, 95% CI 2–3.57, *p* < 0.001) (Figure 1).

**FIGURE 2:**
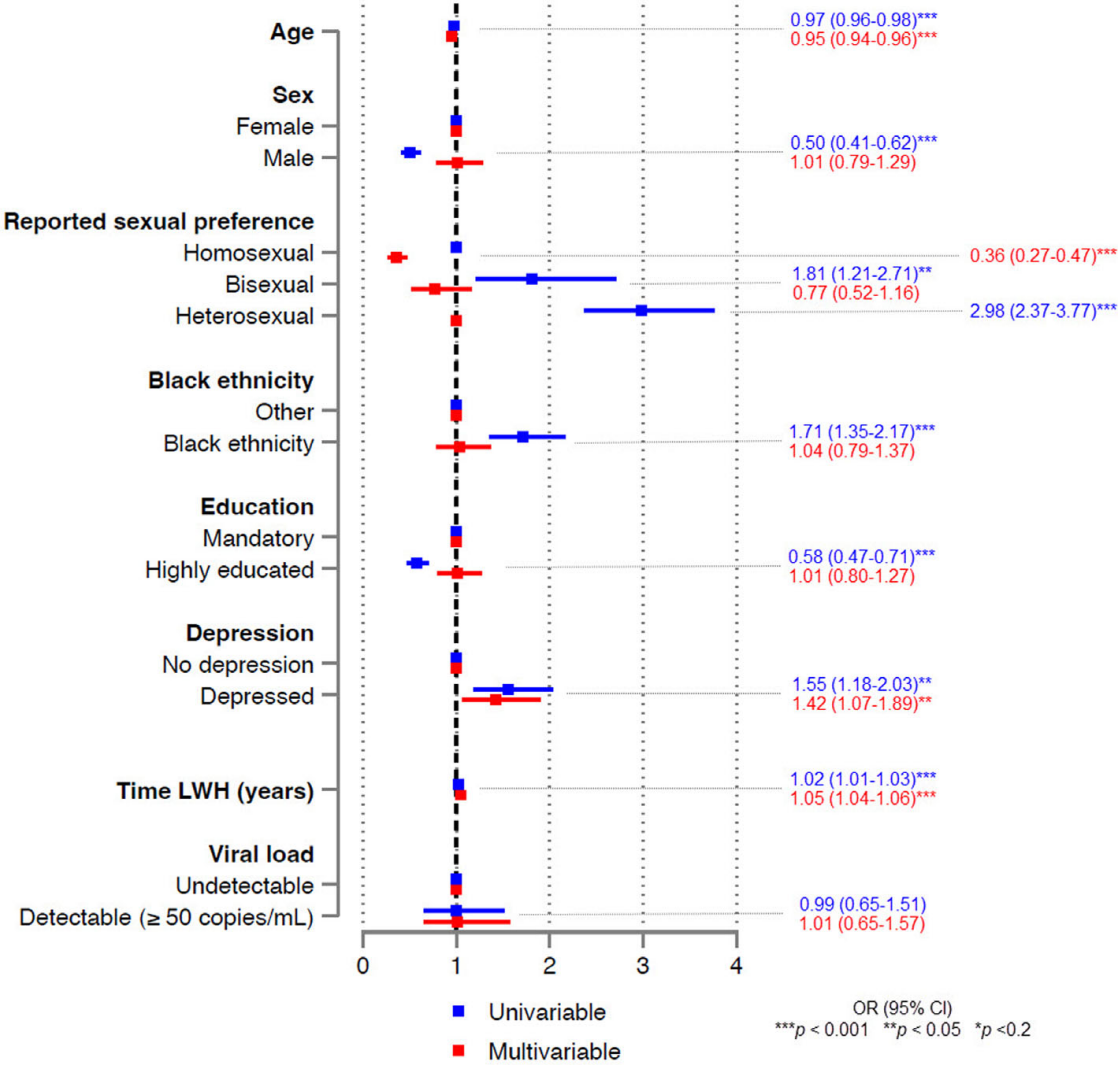
Forest plot of factors associated with HIV diagnosis influence on family planning: Univariate and multivariate logistic regressions with odd ratios and 95% confidence intervals. OR, odd ratio; 95% CI, 95% confidence intervals; *p, p*‐value.

Regarding HIV diagnosis influence on family planning, the multivariable model included age, sex, sexual preference, ethnicity, education, depression, time since diagnosis and actual viral load. It was statistically significant (*n* = 2974, Χ^2^ (9) = 220.72, *p* < 0.001) and explained 8% of the total variance. HIV diagnosis influence on family planning was reduced in older participants (OR 0.95, 95% CI 0.94–0.96, *p* < 0.001) and MSM (OR 0.36, 95% 0.27–0.47, *p* < 0.001) while increased influence was associated with depression (OR 1.42, 95% CI 1.07–1.89, *p* = 0.01) and longer time since HIV diagnosis (OR 1.05, 95% CI 1.04–1.06, *p* < 0.001) (Figure 2).

**FIGURE 3:**
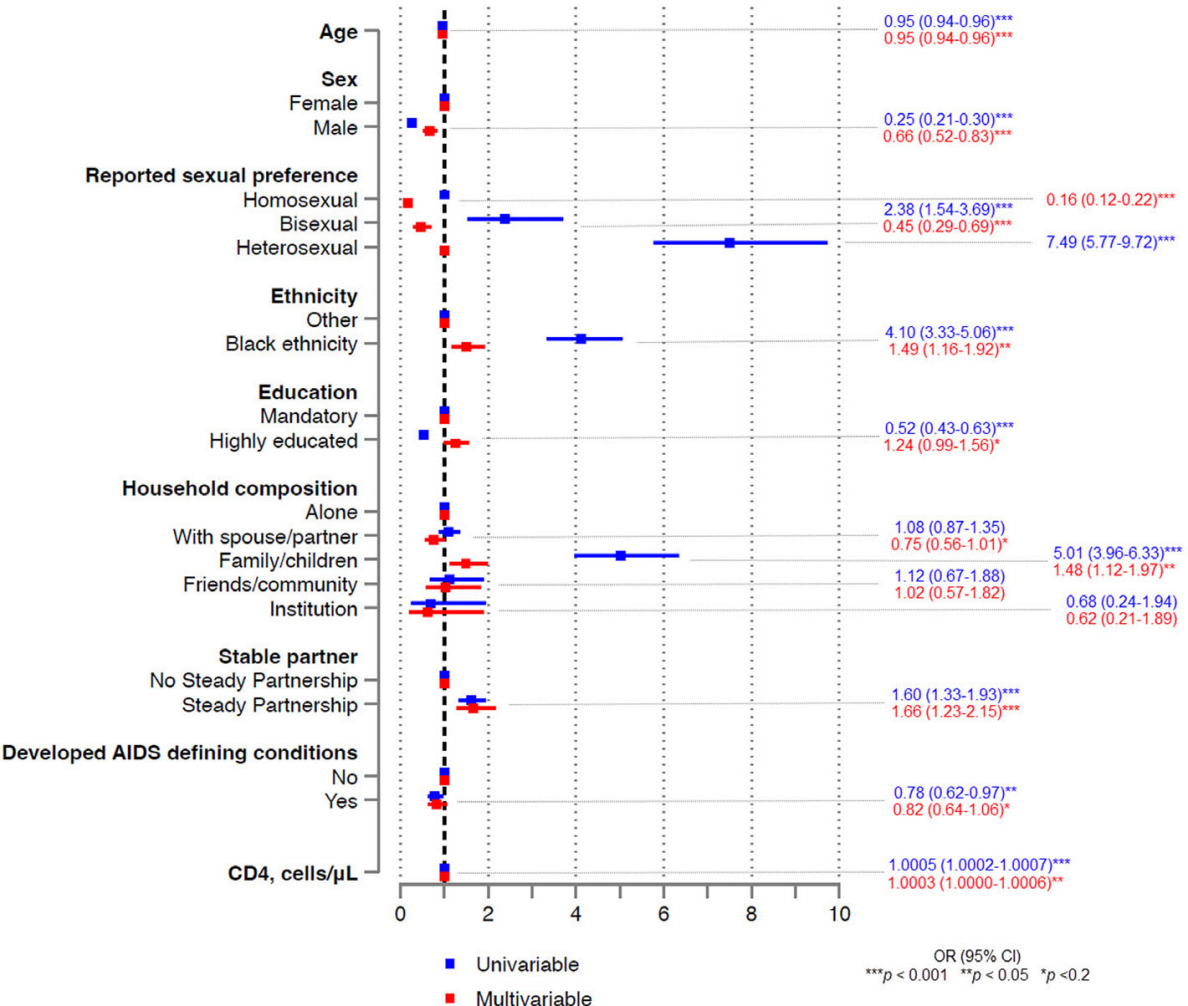
Forest plot of factors associated with discussed parenthood with an HIV physician: Univariate and multivariate logistic regressions with odd ratios and 95% confidence intervals. OR, odd ratio; 95% CI, 95% confidence intervals; *p, p*‐value.

Age, sex, sexual preference, ethnicity, education, employment, living situation and relationship status, as well as the presence of any AIDS defining illness were significantly associated with the likelihood of discussing parenthood with an HIV physician. In the multivariable model, these factors demonstrated a strong fit, accounting for 20.4% of the variance (*n* = 2974, Χ^2^ (13) = 626.60, *p* < 0.001). The odds of discussing future parenthood with an HIV physician decreased with older age (OR 0.95, 95% CI 0.94–0.96, *p* < 0.001) and were lower among heterosexual male participants (OR 0.66, 95% CI 0.52–0.83, *p* < 0.001), MSM (OR 0.16, 95% CI 0.12–0.22, *p* < 0.001) and bisexual participants (OR 0.45, 95% CI 0.29–0.69, *p* < 0.001). The odds were higher with Black ethnicity (OR 1.49, 95% CI 1.16–1.92, *p* = 0.002), living in a family with a child (OR 1.48, 95% CI 1.12–1.97, *p* = 0.006) and being in a stable relationship (OR 1.66, 95% CI 1.23–2.15, *p* < 0.001) (Figure 3).

## DISCUSSION

In this cohort of 2974 PLWH with a median age of 54 years, who were mostly White (75%), 38.7% already had at least one child, while 17.3% expressed parenting desire. Parenting desire was associated with being male and of Black ethnicity. HIV diagnosis somewhat or significantly influenced family planning in 17% of participants overall and in 34.9% of participants expressing parenting desire. Ever having discussed future parenthood with an HIV physician was reported in 21.2% of all participants and only 6.8% of MSM participants. In the multivariate analysis, older age and being MSM were two factors associated with lower parenting desire, less HIV diagnosis influence on family planning and less discussion of future parenthood with an HIV physician.

Our population expressed a lower rate of parenting desire than that observed in previous studies. In a sample of 150 PLWH followed between 2015 and 2016 in a Seattle HIV clinic, 28.7% of participants were considered of reproductive age (<45 years old) and expressed parenting desire [21]. The observed difference could be explained by our participants' older age. Indeed, interest in parenthood increased to 22% among participants aged below 60 years old in our study. A meta‐analysis of 50 studies published between 2000 and 2019 found that parenting desire to be higher among PLWH who were younger than 30 years old, were men, on ART, educated, married or cohabiting and who were childless [22]. The authors suggested that having a partner provides support for child rearing and that parenting desire is influenced by the concept of an ideal family size. Interestingly, our participants who were married or living with a partner had low parenting desire, like participants who already had children. The same meta‐analysis suggested that higher educational attainment, potentially coupled with a higher income, resulted in a better understanding of transmission risks and improved access to medical care, thereby increasing parenting desire [22]. However, our results indicated the opposite, with higher‐educated participants reporting lower desire.

Among male participants, higher rates of parenting desire were observed in heterosexual men (21%) compared to MSM (13.4%). In multivariable analysis, male sex increased parenting desire, while being MSM reduced it. This trend is consistent with the results of a cross‐sectional study recruiting MLWH aged over 18 years and living in Ontario, Canada. The peer interviewer‐administered survey was conducted with 276 men, of whom 158 were MSM. Fatherhood was valued by 69% of participants and 45% expressed an interest in becoming a father in the future [10]. Though participants in the Canadian study were considerably younger than ours, with 93% under 60 years old, a greater interest was observed among heterosexual men (57%) than among MSM (34%). The authors suggested that the heteronormativity surrounding fatherhood may influence both the importance attributed to it and the willingness of MSM to report such a desire [10].

An HIV diagnosis can be experienced as a biographical disruptor, altering self‐identity and life plans. The belief of not being able to procreate can further undermine an individual's concept of self, particularly in cultures where fertility defines womanhood and manhood [11, 12, 23]. The reproductive behaviour of PLWH is shaped by the tension between societal expectations regarding parenthood and the fear of stigma and discrimination associated with their serostatus [11]. Stigma, both perceived and enacted, leads many women living with HIV (WLWH) to renounce plans for motherhood, particularly due to fear of discrimination from healthcare providers and disapproval within their families [23]. In high‐income countries, only one third of WLWH report a pregnancy after HIV diagnosis [23]. In our study, HIV diagnosis influenced family planning for 34.9% of participants who desired parenthood, and women felt more influenced than men. HIV diagnosis influence increased among participants with depression and with longer time since HIV diagnosis. In contrast, older participants and MSM appeared to be less influenced by their HIV diagnosis, possibly because of a lower parenting desire or because their age or sexuality removed them from social expectations relating to parenthood.

Discussing parenting desires and family planning is not universal between PLWH and their healthcare providers. Anticipated stigma may discourage PLWH from seeking information from healthcare professionals [11], who, in turn, may be more inclined to discuss contraception than future parenthood [23]. Thomson et al. found that barely a third of the 150 PLWH seen at a Seattle HIV clinic had discussed parenting desires as initiated by their healthcare provider, with women reporting more such discussions than men (34.6% versus 22.2%) [21]. Fewer than a quarter of our participants recalled ever discussing parenthood with their physician. The timing and initiator of these discussions remain unknown. Participants of Black ethnicity, those living in a family with a child and those in stable relationships were more likely to discuss parenthood compared to older participants, heterosexual men and MSM. Indeed, only 6.8% of MSM had discussed parenthood. Consistent with this, a literature review highlighted that parenthood discussions in the context of HIV occur almost exclusively in heterosexual relationships [24]. Healthcare professionals' assumptions regarding heterosexual men and MSM living with HIV may impede parenthood discussions [14, 25].

Discussing parenthood is an opportunity to deliver counselling on both contraception and conception [21]. Irrespective of gender, sexuality, origin, or parenting desire, acknowledging the possibility of conceiving may have positive effects on the well‐being and self‐esteem of PLWH, optimising understanding of U=U and reducing internalised and anticipated stigma [12, 23]. The implementation of HIV fertility programmes with patient‐centred counselling sessions and discrimination reduction interventions in the community has been described [11, 26]. HIV support workers may help PLWH in accessing adoption and fertility services, helping them navigate institutionalised and perceived stigma [26]. Peer support groups are an effective way for PLWH to acquire information on parenthood, combat discrimination and become empowered [11].

Our study has limitations. By setting the study only in university or cantonal hospitals, the participants may not be fully representative of all PLWH in Switzerland. Future studies to include other centres could be designed using a shorter questionnaire which could be completed in less time or online, or else by incentivizing these centres to accommodate time for questionnaire completion. As there was no validated tool that fully matched our study objectives, we developed a questionnaire with input from HIV physicians and community members. However, the structure of the questionnaire may have favoured participants with fewer children as a new section had to be opened for each child for participants with >1 child (Supplementary material 1). Questionnaire responses may have been subject to recall or social desirability bias, as they were completed with the assistance of the participants' HIV physician. As the questionnaire consisted of closed questions, only one of which explored HIV influence, it was not possible to study the influence of U = U on parenting desire. This cross‐sectional design also limits interpretation to associations rather than causality. Finally, ‘I would like to become a parent in the future’ was not defined, allowing for various interpretations—as a wish, interest, or considered intention to have children—which may have influenced responses. Against these limitations, our study has explored parenting desire in a European high‐income country in the U=U era.

## CONCLUSION

In this sample of PLWH, a minority expressed parenting desire, possibly due to high median participant age and 17% felt that HIV influenced their family planning decisions. While female participants were more likely to have discussed future parenthood than male participants, there was a further disparity between heterosexual men and MSM. Whilst surrogacy is prohibited in Switzerland, MSM may have access to parenthood in other ways. Our findings suggest that there is room for HIV physicians to proactively invite parenting discussions with all PLWH.

## AUTHOR CONTRIBUTIONS

KEAD, MC and CM conceptualised the study. KK was in charge of the online survey and extracting the data. All statistical analyses were performed by JD. CM wrote the manuscript draft and KEAD revised it and prepared it for publication. All authors reviewed the final manuscript.

## MEMBERS OF THE SWISS HIV COHORT STUDY

Abela IA, Aebi‐Popp K, Anagnostopoulos A, Bernasconi E, Braun DL, Bucher HC, Calmy A, Cavassini M (Chairman of the Clinical and Laboratory Committee), Ciuffi A, Dollenmaier G, Egger M, Elzi L, Fehr JS, Fellay J, Frigerio Malossa S, Fux CA, Günthard HF, Hachfeld A, Haerry DHU (deputy of “Positive Council”), Hasse B, Hirsch HH, Hoffmann M, Hösli I, Huber M, Jackson‐Perry D (patient representatives), Kahlert CR, Kaufmann D, Keiser O, Klimkait T, Kouyos RD, Kovari H, Kusejko K (Head of Data Centre), Labhardt ND, Leuzinger K, Martinez de Tejada B, Marzolini C, Metzner KJ, Müller N, Nemeth J, Nicca D, Notter J, Paioni P (Chairman of the Mother & Child Substudy), Pantaleo G, Perreau M, Rauch A (President of the SHCS), Salazar‐Vizcaya LP, Schmid P, Segeral O, Speck RF, Stöckle M, Surial B, Tarr PE, Trkola A, Wandeler G (Chairman of the Scientific Board), Weisser M, Yerly S.

## FUNDING INFORMATION

This study has been financed within the framework of the SHCS, supported by the Swiss National Science foundation (grant #33FI‐0‐229 621), by SHCS project P896 and by the SHCS research foundation.

## CONFLICT OF INTEREST STATEMENT

CM has received sponsorship to attend a specialist conference from Gilead Sciences. IAA received an advanced physician scientist grant from UMZH. MC's institution has received research grants from Gilead, travel grants from Gilead and MSD and expert opinion fees from Gilead, MSD, ViiV. KEAD's institution has received project grants, sponsorship to attend specialist meetings and honoraria for presentations from Gilead Sciences, fees for expert opinion panels from MSD, and sponsorship for educational events from Gilead, MSD and ViiV. The other authors have no competing interests to declare.

## ETHICS STATEMENT

The Swiss HIV Cohort Study (SHCS) was approved by the local ethics committees of all participating study sites. All participants in the SHCS gave informed consent.

## Supporting information


**Appendix S1.** Questionnaire on CHildren Of the Cohort (CHOC).

**Figure S1.** Parenting desire by reported sex preference.
**Figure S2.** Parenting desire by age category and ethnicity.
**Figure S3.** HIV diagnosis influence on family planning by reported sex preference.
**Figure S4.** HIV diagnosis influence on family planning by age category and ethnicity.
**Figure S5.** Discussed parenthood with an HIV physician by reported sex preference.
**Figure S6.** Discussed parenthood with an HIV physician by age category and ethnicity.

## Data Availability

Data are available to be shared upon reasonable request.

## References

[1] Slogrove AL , Powis KM , Johnson LF , Stover J , Mahy M . Estimates of the global population of children who are HIV‐exposed and uninfected, 2000‐18: a modelling study. Lancet Glob Health. 2020;8(1):e67‐e75. doi:10.1016/S2214-109X(19)30448-6 31791800 PMC6981259

[2] Aebi‐Popp K , Bernasconi E , Kahlert C , et al. Recommendations of the Swiss Federal Commission for Sexual Health (FCSH) for Medical Care of HIV‐Infected Women and their Offspring. Vol. 2018. Bulletin OFSP; 2018:10. doi:10.13140/RG.2.2.21470.08004/2

[3] Wagner N , Crisinel PA , Kahlert C , Martinez De Tejada B . Breastfeeding for HIV‐positive mothers in Switzerland : are we ready to discuss ? Rev Med Suisse. 2020;16(712):2050‐2054. 33112519

[4] Vernazza P , Hirschel B , Bernasconi E , Flepp M . HIV positive individuals not suffering from any other STD and adhering to an effective antiretroviral treatment do not transmit HIV sexually. Bull Méd Suisses. 2008;89(5):165‐169.

[5] Cohen MS , McCauley M , Gamble TR . HIV treatment as prevention and HPTN 052. Curr Opin HIV AIDS. 2012;7(2):99‐105. doi:10.1097/COH.0b013e32834f5cf2 22227585 PMC3486734

[6] Rodger AJ , Cambiano V , Bruun T , et al. Sexual activity without condoms and risk of HIV transmission in Serodifferent couples when the HIV‐positive partner is using suppressive antiretroviral therapy. JAMA. 2016;316(2):171‐181. doi:10.1001/jama.2016.5148 27404185

[7] Rodger AJ , Cambiano V , Bruun T , et al. Risk of HIV transmission through condomless sex in serodifferent gay couples with the HIV‐positive PARTNER taking suppressive antiretroviral therapy (PARTNER): final results of a multicentre, prospective, observational study. Lancet. 2019;393(10189):2428‐2438. doi:10.1016/S0140-6736(19)30418-0 31056293 PMC6584382

[8] Adilo TM , Wordofa HM . Prevalence of fertility desire and its associated factors among 15‐ to 49‐year‐old people living with HIV/AIDS in Addis Ababa, Ethiopia: a cross‐sectional study design. HIV AIDS (Auckl). 2017;9:167‐176. doi:10.2147/HIV.S133766 28919821 PMC5587090

[9] Ogilvie GS , Palepu A , Remple VP , et al. Fertility intentions of women of reproductive age living with HIV in British Columbia, Canada. AIDS. 2007;21(Suppl 1):S83‐S88. doi:10.1097/01.aids.0000255090.51921.60 17159593

[10] Kyne LT , Yudin MH , Bekele T , et al. Understanding the importance of fatherhood among men living with HIV in Ontario. J Int Assoc Provid AIDS Care. 2021;20:23259582211016133. doi:10.1177/23259582211016133 34000889 PMC8135195

[11] Pollard R , Saleem H . Reproductive identities following an HIV diagnosis: strategies in the face of biographical disruption. Cult Health Sex. 2020;22(4):385‐397. doi:10.1080/13691058.2019.1603399 31012809

[12] Pralat R , Burns F , Anderson J , Barber TJ . Can HIV‐positive gay men become parents? How men living with HIV and HIV clinicians talk about the possibility of having children. Sociol Health Illn. 2021;43(2):281‐298. doi:10.1111/1467-9566.13218 33222191 PMC8170559

[13] Hachfeld A , Atkinson A , Calmy A , et al. Decrease of condom use in heterosexual couples and its impact on pregnancy rates: the Swiss HIV cohort study (SHCS). HIV Med. 2022;23(1):60‐69. doi:10.1111/hiv.13152 34476886 PMC9290944

[14] Yudin MH , Kennedy VL , Bekele T , et al. An exploration of the fertility desires and intentions of men living with HIV in Ontario, Canada. AIDS Care. 2021;33(2):262‐272. doi:10.1080/09540121.2020.1734175 32164422

[15] Confédération Suisse . Loi fédérale sur la procréation médicalement assistée (LPMA, RS 810.11) [Loi fédérale]. Berne: Fedlex. 1998 Accessed September 25, 2025. https://www.fedlex.admin.ch/filestore/fedlex.data.admin.ch/eli/cc/2000/554/20250801/fr/pdf‐a/fedlex‐data‐admin‐ch‐eli‐cc‐2000‐554‐20250801‐fr‐pdf‐a.pdf

[16] Confédération Suisse . Ordonnance sur la procréation médicalement assistée (OPMA, RS 810.111.1) [Ordonnance fédérale]. Berne: Fedlex. 2000 Accessed September 25, 2025 https://www.fedlex.admin.ch/eli/cc/2001/214/fr

[17] Confédération Suisse . Code civil suisse (CC, RS 210) [Loi fédérale]. Berne: Lawbrary. 2024 Accessed September 9, 2025. https://lawbrary.ch/gesetz/210/CC/v2024.01/fr/art120/code‐civil‐suisse/art‐120/

[18] Scherrer AU , Traytel A , Braun DL , et al. Cohort profile update: the Swiss HIV cohort study (SHCS). Int J Epidemiol. 2022;51(1):33‐34j. doi:10.1093/ije/dyab141 34363666

[19] Merlin C , Damas J , Tshikung ON , et al. P246 : CHildren Of the Cohort (CHOC) study : The unseen population behind people living with HIV [Poster]. 2024.

[20] Nguyen H , Hampel B , Garcia Nuñez D , et al. Identifying and characterizing trans women in the Swiss HIV cohort study as an epidemiologically distinct risk group. Clin Infect Dis. 2021;74(8):1468‐1475. doi:10.1093/cid/ciab628 PMC9049251 34282827

[21] Thomson KA , Dhanireddy S , Andrasik M , et al. Fertility desires and preferences for safer conception strategies among people receiving care for HIV at a publicly‐funded clinic in Seattle, WA. AIDS Care. 2018;30(1):121‐129. doi:10.1080/09540121.2017.1390541 29067843 PMC6117831

[22] Yan X , Du J , Ji G . Prevalence and factors associated with fertility desire among people living with HIV: a systematic review and meta‐analysis. PLoS One. 2021;16(3):e0248872. doi:10.1371/journal.pone.0248872 33735265 PMC7971888

[23] Huertas‐Zurriaga A , Palmieri PA , Edwards JE , et al. Motherhood and decision‐making among women living with HIV in developed countries: a systematic review with qualitative research synthesis. Reprod Health. 2021;18(1):148. doi:10.1186/s12978-021-01197-6 34246286 PMC8272303

[24] Martins A , Alves S , Chaves C , Canavarro MC , Pereira M . Prevalence and factors associated with fertility desires/intentions among individuals in HIV‐serodiscordant relationships: a systematic review of empirical studies. J Int AIDS Soc. 2019;22(5):e25241. doi:10.1002/jia2.25241 31099170 PMC6523008

[25] Pralat R , Anderson J , Burns F , Yarrow E , Barber TJ . Discussing parenthood with gay men diagnosed with HIV: a qualitative study of patient and healthcare practitioner perspectives. BMC Public Health. 2021;21(1):2300. doi:10.1186/s12889-021-12285-4 34923967 PMC8684690

[26] Cane TPC . Facilitating and supporting HIV+ parenthood: lessons for developing the advocate role of voluntary HIV support services workers. Sex Reprod Healthc. 2018;16:186‐191. doi:10.1016/j.srhc.2018.04.001 29804765

