# Supplementary material for: CHildren of the Cohort Study (CHOC): Exploring parenting desire among people living with HIV in Switzerland
Source: HIV Med. 2026 May 11;27(8):1287–97. doi: 10.1111/hiv.70253 (PMC13432503; doi:10.1111/hiv.70253)
Supplement: Supplementary file 1 — Appendix S1. Questionnaire on CHildren Of the Cohort (CHOC). [file HIV-27-1287-s001.docx]

**Appendix 1: Questionnaire on CHildren Of the Cohort (CHOC)**

To HIV physician completing the questionnaire with participants:

Would you like to complete the CHildren Of the Cohort (CHOC) questionnaire now? (yes/no)

**WARNING: *Please note that you cannot return to the CHOC survey page once you submit the CHOC questionnaire. If you need to re-open the survey page, you can. However, any previously submitted data will be lost.***

**Study questionnaire:**

We are interested in knowing how many children our patients have, including children born prior to the parents’ HIV diagnosis, and what part children play in our patients’ lives. We would be most grateful if you could reply to the following questions:

Choose language (English, French, German, Italian, Portuguese or Spanish)

Do you have any children?

Yes:

1. How many? (number)

**Questions to be completed for each child:**

1. Year of birth? (drop-down list from 1960-2022)
2. Country of birth? (drop-down list)
3. HIV status? (positive / negative / unknown)
4. Does the child live with you? (yes/no)
5. If the child does not live with you, where does the child live? (drop-down list with ‘not applicable’ for patients answering ‘yes’ to Q5)
6. Do you support the child economically? (yes/no)
7. Are you in regular contact with the child (at least once a month)? (yes/no)
8. Does the child know about your HIV status? (yes/no/don’t know)
9. Does the child’s other parent know about your HIV status? (yes/no/don’t know)

**Questions to be answered once (rather than for each child):**

1. With respect to this statement: ‘I would like to become a parent in the future?’, do you strongly agree, agree, disagree or strongly disagree? (4-point Likert scale)
2. How much does your HIV diagnosis influence family planning? (a lot / a bit / not really / not at all) (4-point Likert scale)
3. Have you ever discussed being a parent with your treating HIV doctor? (yes/no)
4. How many grandchildren do you have? (number)

Thank you very much for completing this questionnaire.

- Submit

No:

1. With respect to this statement: ‘I would like to become a parent in the future?’, do you strongly agree, agree, disagree or strongly disagree? (4-point Likert scale)
2. How much does your HIV diagnosis influence family planning? (a lot / a bit / not really / not at all) (4-point Likert scale)
3. Have you ever discussed being a parent with your treating HIV doctor? (yes/no)

Thank you very much for completing this questionnaire.

- Submit
