# Supplementary material for: CHildren of the Cohort Study (CHOC): Exploring parenting desire among people living with HIV in Switzerland
Source: HIV Med. 2026 May 11;27(8):1287–97. doi: 10.1111/hiv.70253 (PMC13432503; doi:10.1111/hiv.70253)
Supplement: Supplementary file 2 — Figure S1. Parenting desire by reported sex preference. Figure S2. Parenting desire by age category and ethnicity. Figure S3. HIV diagnosis influence on family planning by reported sex preference. Figure S4. HIV diagnosis influence on family planning by age category and ethnicity. Figure S5. Discussed parenthood with an HIV physician by reported sex preference. Figure S6. Discussed parenthood with an HIV physician by age category and ethnicity. [file HIV-27-1287-s002.docx]

Figure legends

Figure 1: Parenting desire by reported sex preference

Figure 2: Parenting desire by age category and ethnicity

Figure 3: HIV diagnosis influence on family planning by reported sex preference

Figure 4: HIV diagnosis influence on family planning by age category and ethnicity

Figure 5: Discussed parenthood with an HIV physician by reported sex preference

Figure 6: Discussed parenthood with an HIV physician by age category and ethnicity


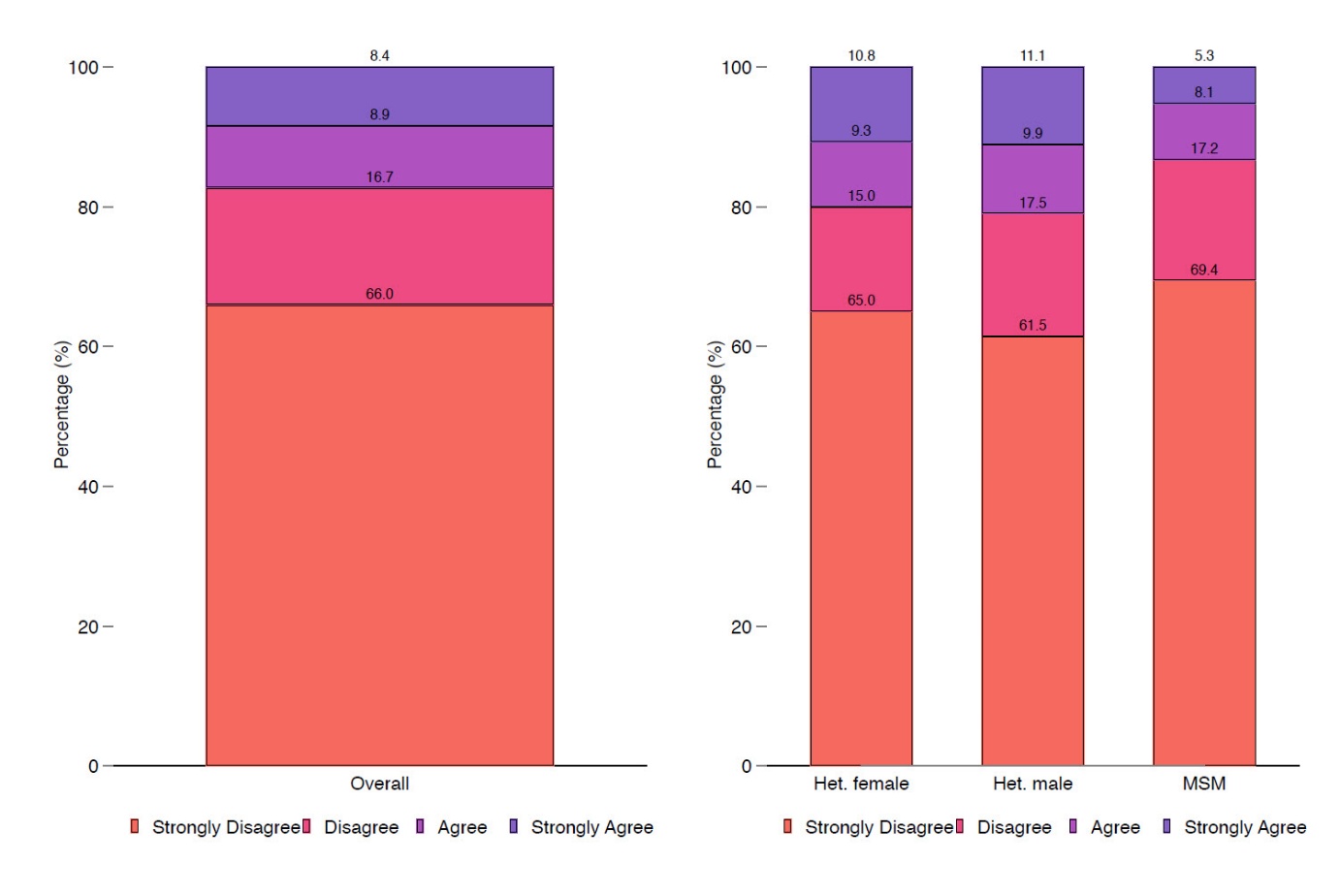


Figure 1: Parenting desire by reported sex preference.

n=808 heterosexual female (27.17%), n=838 heterosexual male (28.18%); n=1328 male having sex with male (MSM) (44.65%).


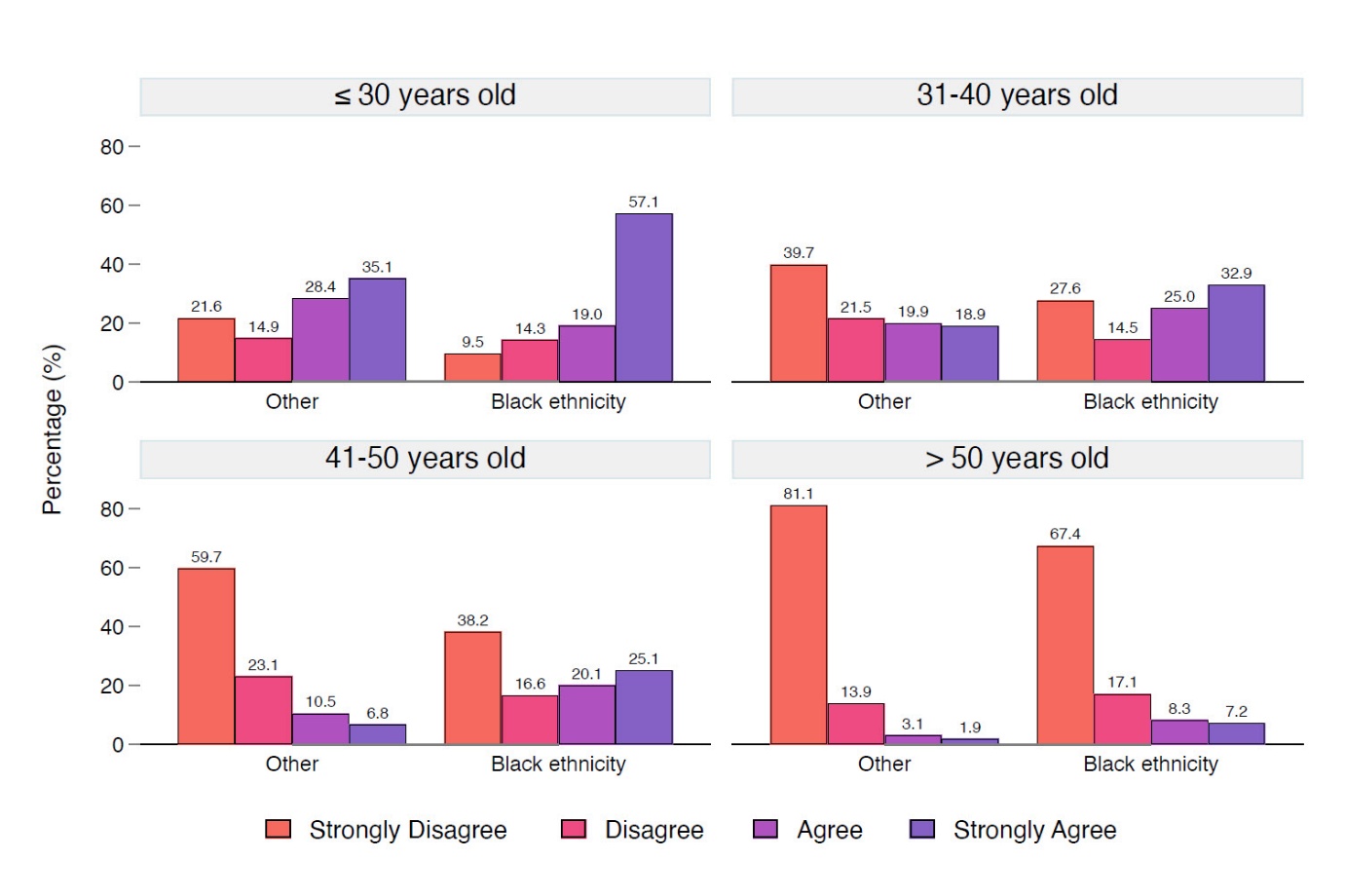


Figure 2: Parenting desire by age category and ethnicity.

n=95 participants < 30 years old (3.19%), n=388 participants 31-40 years old (13.05%), n= 715 participants 41-50 years old (24.04%), n=1776 participants >50 years old (59.72%).


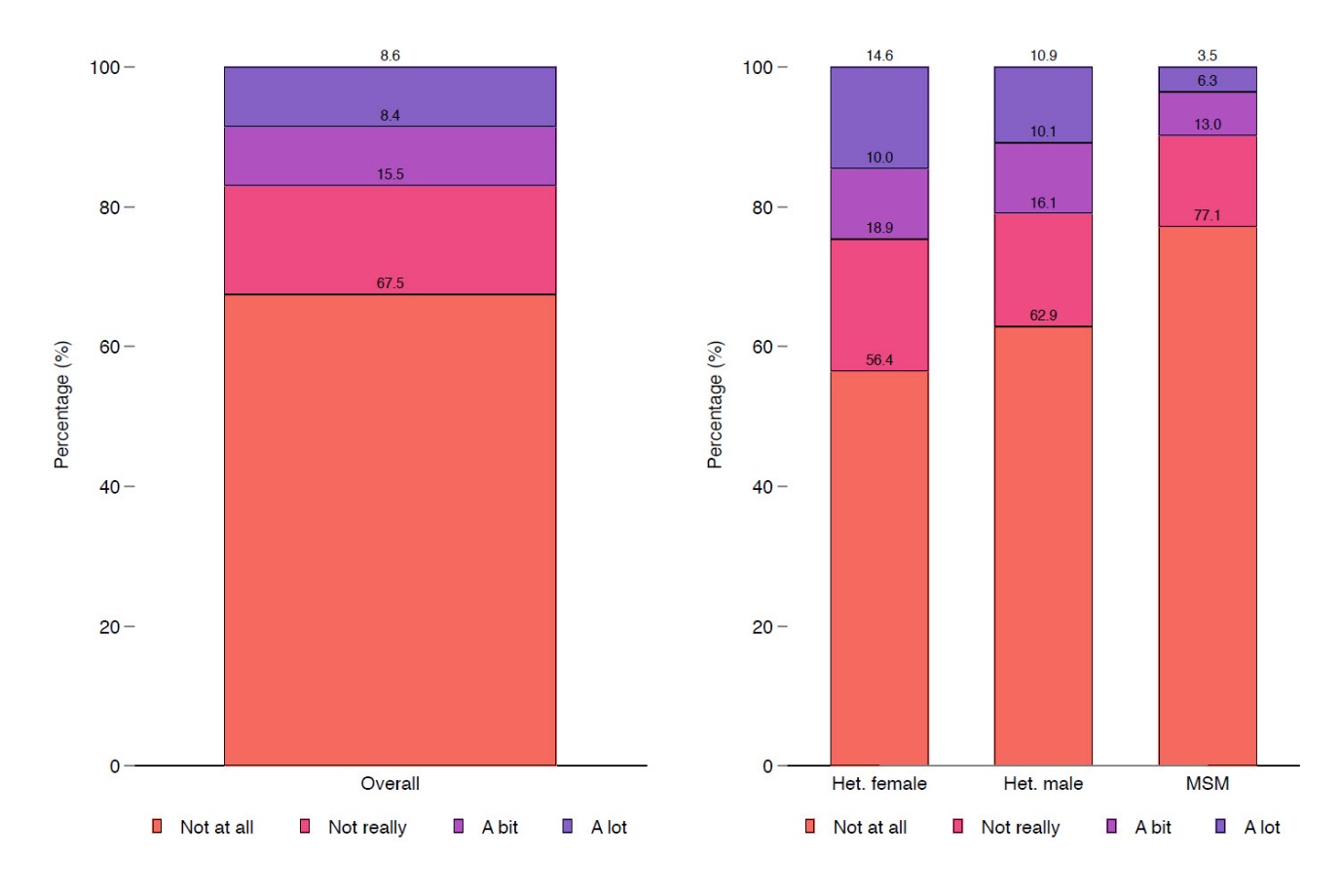


Figure 3: HIV diagnosis influence on family planning by reported sex preference

n=808 heterosexual female (27.17%), n=838 heterosexual male (28.18%); n=1328 male having sex with male (MSM) (44.65%).


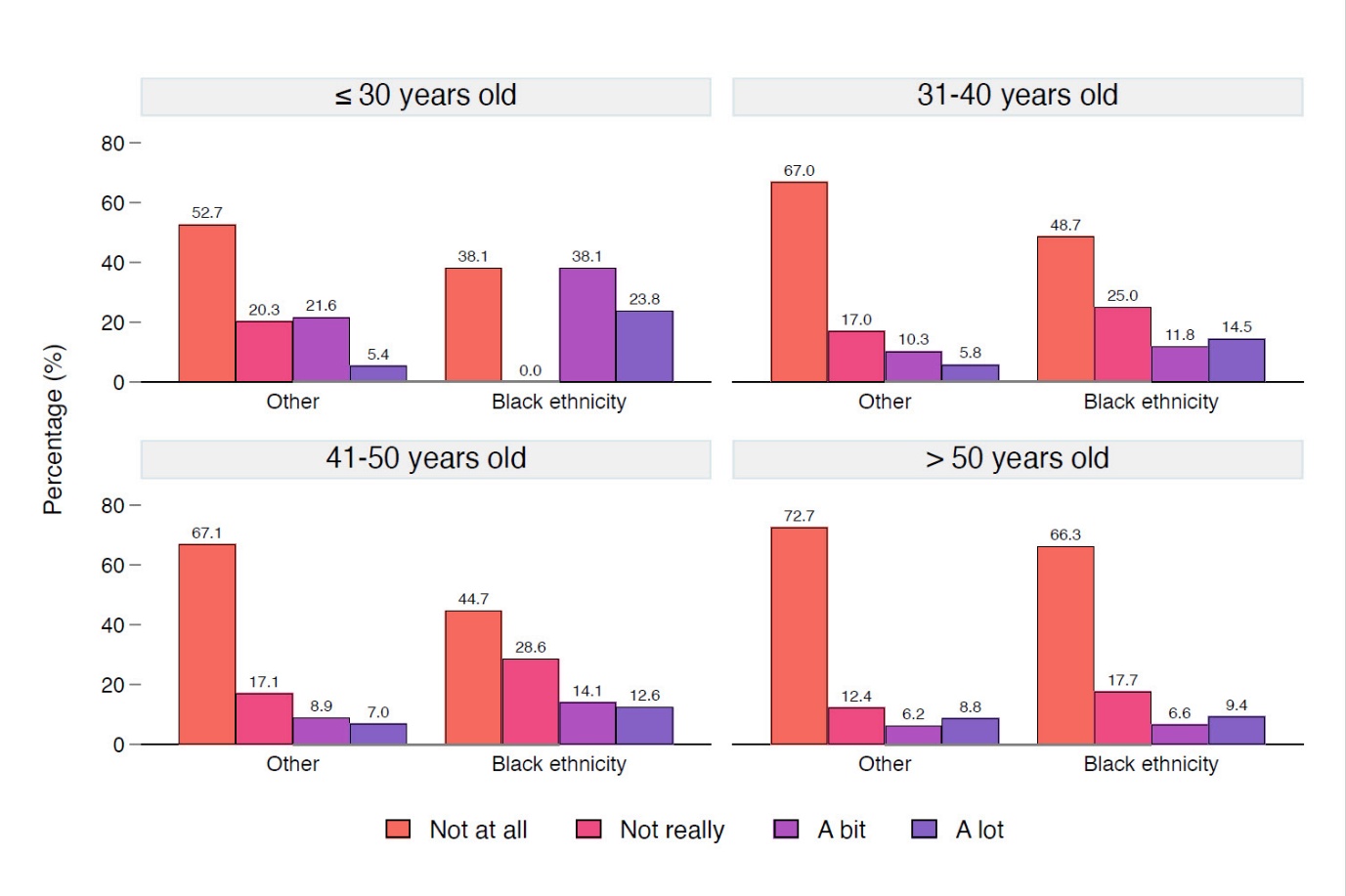


Figure 4: HIV diagnosis influence on family planning by age category and ethnicity

n=95 participants < 30 years old (3.19%), n=388 participants 31-40 years old (13.05%), n= 715 participants 41-50 years old (24.04%), n=1776 participants >50 years old (59.72%).


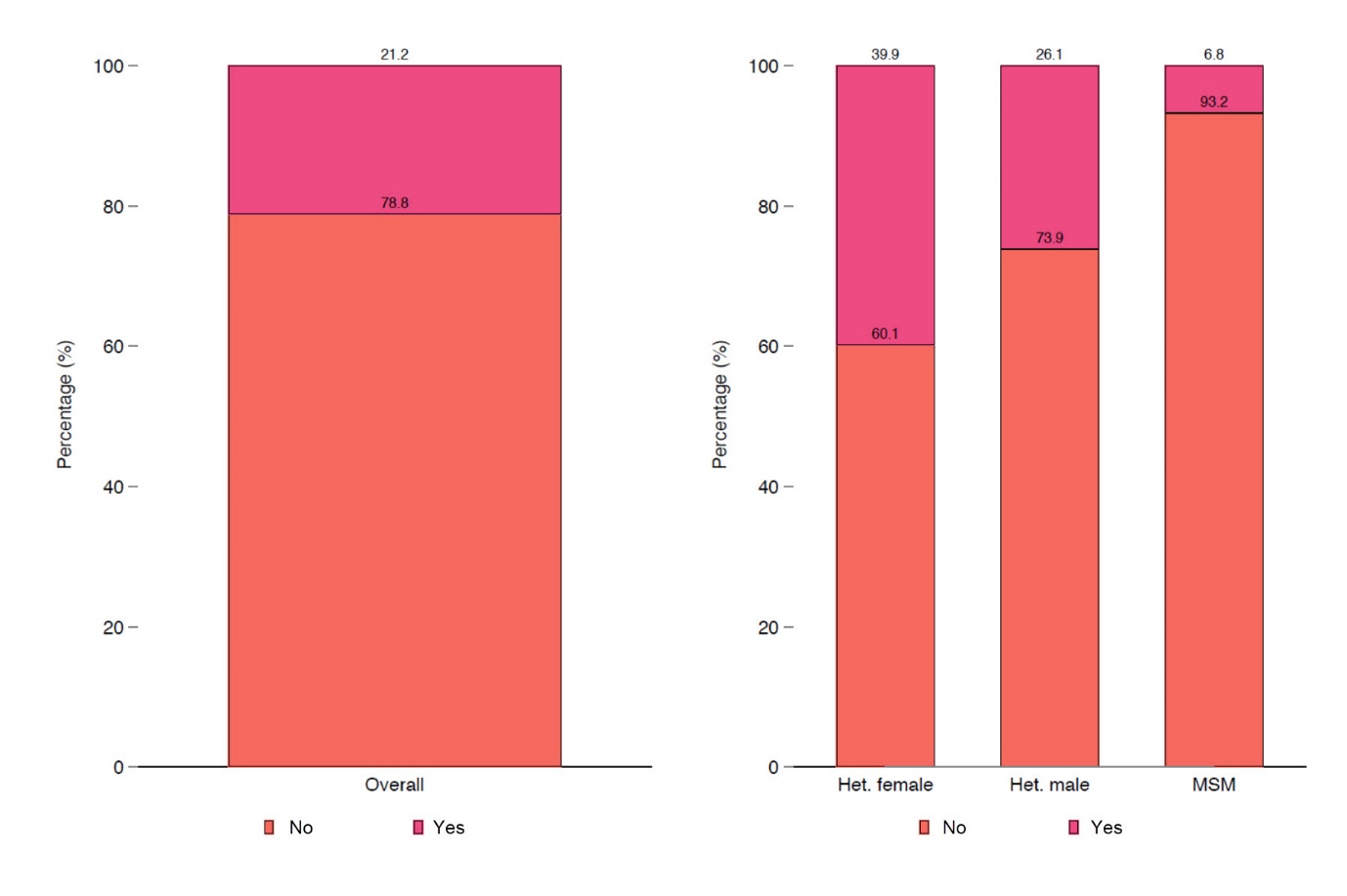


Figure 5: Discussed parenthood with an HIV physician by reported sex preference

n=808 heterosexual female (27.17%), n=838 heterosexual male (28.18%); n=1328 male having sex with male (MSM) (44.65%).


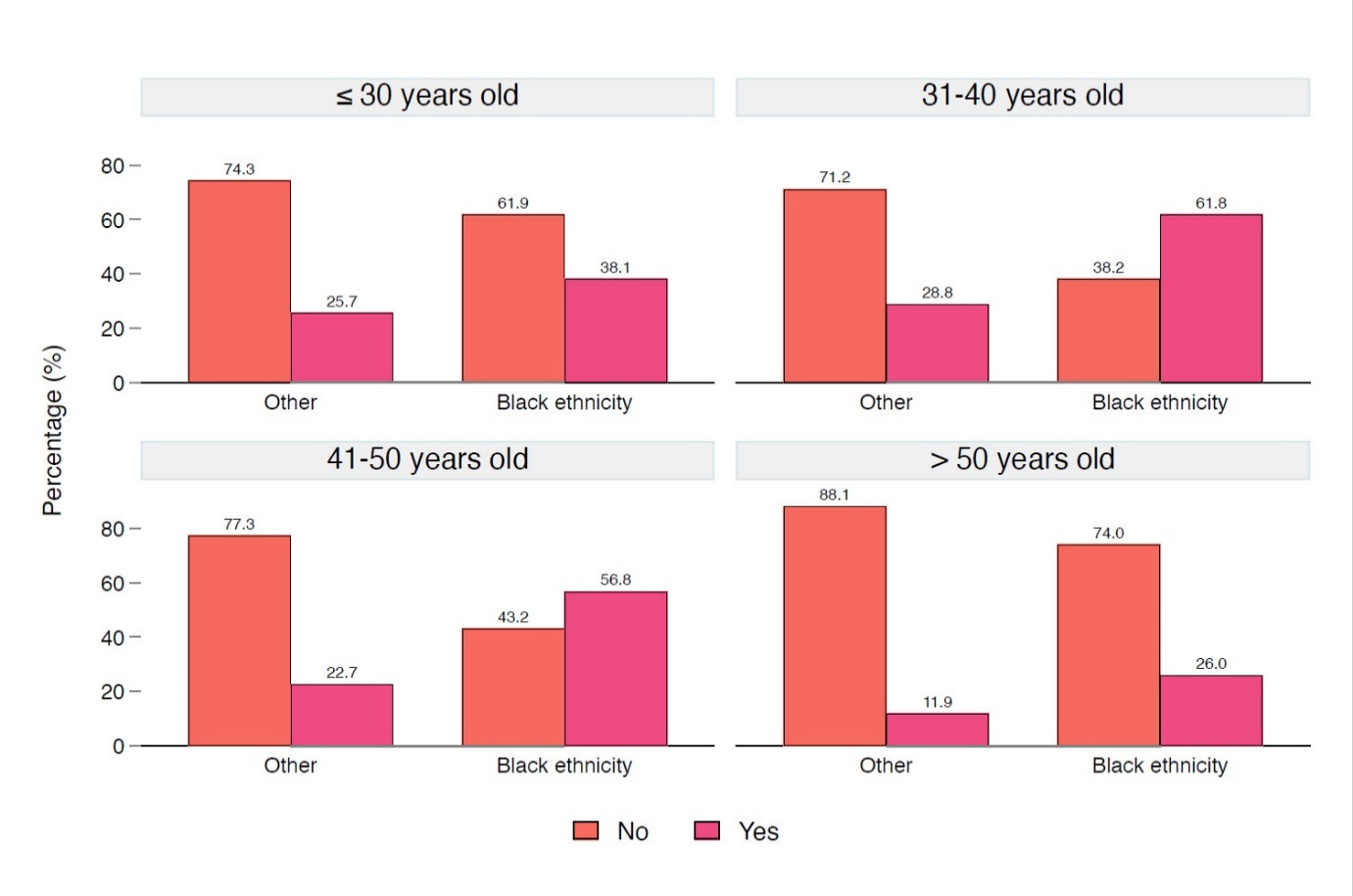


Figure 6: Discussed parenthood with an HIV physician by age category and ethnicity

n=95 participants < 30 years old (3.19%), n=388 participants 31-40 years old (13.05%), n= 715 participants 41-50 years old (24.04%), n=1776 participants >50 years old (59.72%).
